# Immunomodulation Therapy to Treat Severe Malaria With Multiple Organ Failure

**DOI:** 10.1097/CCE.0000000000001471

**Published:** 2026-08-27

**Authors:** Saige Camara, Amy Buck, Mikayla Burrell, H. David Humes, Stuart L. Goldstein, Carlie N. Myers, Michael Humes

**Affiliations:** 1 Division of Critical Care, Cincinnati Children’s Hospital Medical Center, Cincinnati, OH.; 2 Division of Nephrology and Hypertension, Cincinnati Children’s Hospital Medical Center, Cincinnati, OH.; 3 Division of Nephrology, Department of Internal Medicine, University of Michigan Medical Center, Ann Arbor, MI.; 4 Innovative BioTherapies, Ann Arbor, MI.; 5 Department of Pediatrics, University of Cincinnati College of Medicine, Cincinnati, OH.

**Keywords:** cytokines, immunomodulation, inflammation, leukocytes, malaria, sepsis

## Abstract

**BACKGROUND:**

Malaria causes more than 600,000 deaths annually. Severe malaria is frequently complicated by acute kidney injury (AKI) and multiple organ failure driven by dysregulated host hyperinflammation. Elevated cytokines, including interleukin-6 and tumor necrosis factor alpha, are associated with disease severity and mortality, yet effective adjunctive immunomodulatory therapies are lacking. We report the first clinical use of the selective cytopheretic device (SCD), an extracorporeal immunomodulatory therapy, in severe malaria.

**CASE SUMMARY:**

A 5-year-old girl returning from the Democratic Republic of the Congo presented with fever, shock, 10.9% parasitemia, AKI, and evolving multiple organ dysfunction. Despite IV artesunate and RBC exchange, she developed refractory shock and severe lactic acidosis. Continuous renal replacement therapy with adjunctive SCD therapy was initiated, after which inflammatory markers, vasopressor requirements, and metabolic acidosis improved with progressive organ recovery.

**CONCLUSIONS:**

Targeted extracorporeal immunomodulation may represent a promising adjunctive strategy for severe malaria.

**KEY POINTS**

**Question**: Could the selective cytopheretic device be the long-sought-after adjunct immunomodulation therapy for treating severe malaria.

**Findings**: Use of a novel immunomodulatory device may have contributed to reversal of severe malaria-associated multiple organ failure and improved clinical outcome in a pediatric patient. This case further reinforces the critical role that hyperinflammation plays in severe malaria and the need to manage this aspect of the disease.

**Meaning**: The selective cytopheretic device may be a key adjunct therapy for treating severe malaria with multiple organ failure

In 2024, an estimated 282 million malaria cases and 610,000 deaths occurred worldwide, with 94% of cases and 95% of deaths concentrated in Africa (1). *Plasmodium falciparum* accounts for over 90% of malaria-related mortality and is uniquely pathogenic due to its ability to induce intravascular hemolysis and endothelial cytoadherence (2–4). Sequestration of infected RBCs within the microvasculature leads to impaired tissue perfusion, lactic acidosis, and multiple organ dysfunction (5–7). Beyond mechanical obstruction, severe malaria (SM) is characterized by a dysregulated systemic inflammatory response that plays a central role in organ failure and mortality (8, 9).

Acute kidney injury (AKI) is a complication of SM and an independent predictor of mortality in children with SM (10). Reported incidence ranges from 20% to 40%, and up to 59% in some pediatric cohorts (11–16). Increasing evidence implicates dysregulated systemic inflammation in the pathogenesis of AKI. Patients with SM demonstrated elevated levels of proinflammatory cytokines, including interleukin (IL)-6 and tumor necrosis factor alpha (TNF-α). Higher IL-6 concentrations have been observed in SM compared with uncomplicated malaria and among nonsurvivors compared with survivors (17). Elevated TNF-α has similarly been associated with organ dysfunction and progression to severe disease (18, 19). The magnitude and imbalance between pro- and anti-inflammatory responses are key determinants of organ failure and mortality, highlighting immunomodulation as a potential therapeutic strategy (20).

Given the central role of dysregulated inflammation in SM, immunomodulatory strategies that target the host response represent a promising adjunct to antiparasitic therapy. The selective cytopheretic device (SCD) is an extracorporeal therapy integrated with hemodialysis that uses a low-shear, low-ionized calcium environment to sequester and reprogram activated neutrophils and monocytes, returning them to circulation in a less proinflammatory state and dampening systemic hyperinflammation. The U.S. Food and Drug Administration approved the SCD under a Humanitarian Device Exemption for pediatric sepsis-associated AKI requiring continuous renal replacement therapy (CRRT) (21). Clinical experience has demonstrated reductions in circulating IL-6 and TNF-α with associated clinical improvement (21–26). Given its established safety profile and biologic rationale, SCD therapy represents a promising adjunctive strategy to mitigate hyperinflammation and improve outcomes in SM.

## PATIENT HISTORY

A 5-year-old girl with a history of developmental delay presented to an outside hospital with a 5-day history of fever, progressive fatigue, and gastrointestinal symptoms, including vomiting, diarrhea, and reduced oral intake, despite prior symptomatic treatment with ondansetron. On examination, she was tachycardic, tachypneic, lethargic, and clinically dehydrated with diffuse abdominal tenderness. Laboratory evaluation revealed leukocytosis (17,400/μL), anemia (hemoglobin 8.8 g/dL), profound thrombocytopenia (4,500/μL), AKI (blood urea nitrogen 76 mg/dL; creatinine 1.89 mg/dL), hyperkalemia (5.9 mmol/L), hyperbilirubinemia (total bilirubin 6.9 mg/dL), transaminitis (aspartate aminotransferase 189 U/L; alanine aminotransferase 99 U/L), and severe lactic acidosis (8 mmol/L). Peripheral blood smear demonstrated intraerythrocytic parasites consistent with malaria. Subsequent elicitation of travel history revealed recent return from the Democratic Republic of Congo 2 weeks prior without malaria chemoprophylaxis. Blood cultures were drawn, and she received IV crystalloid resuscitation and Ceftriaxone before transferring to Cincinnati Children’s Hospital Medical Center (CCHMC).

Upon arrival to the CCHMC emergency department, she appeared critically ill with altered mental status, cool extremities, and a high-grade fever (103.5°F). She was tachycardic with age-appropriate blood pressures, concerning for compensated septic shock. Repeat laboratory studies confirmed prior abnormalities with additional evidence of hemolysis and systemic inflammation. Peripheral smear demonstrated parasitemia of 10.9%; blood cultures remained negative for bacteria. Head CT showed no cerebral edema. She was admitted to the PICU for severe *P. falciparum* malaria complicated by shock and evolving multiple organ dysfunction, with a Pediatric Risk of Mortality III (27) score of 25.

Shortly after admission to the CCHMC PICU, she developed worsening encephalopathy and lactic acidosis. She was intubated on hospital day (HD) 2 and supported with mechanical ventilation. An acute decompensation followed, including hypotension, bradycardia, and brief pulselessness requiring ~45 seconds of cardiopulmonary resuscitation before return of spontaneous circulation. Vasoactive support with epinephrine and vasopressin was initiated. Empiric antimicrobials were broadened to cefepime and vancomycin. Although cerebral malaria was suspected, lumbar puncture was deferred due to patient instability. In consultation with the Centers for Disease Control and Prevention, IV artesunate was requested; pending institutional availability, enteral artemether-lumefantrine was initiated as bridging therapy.

**Figure 1.**
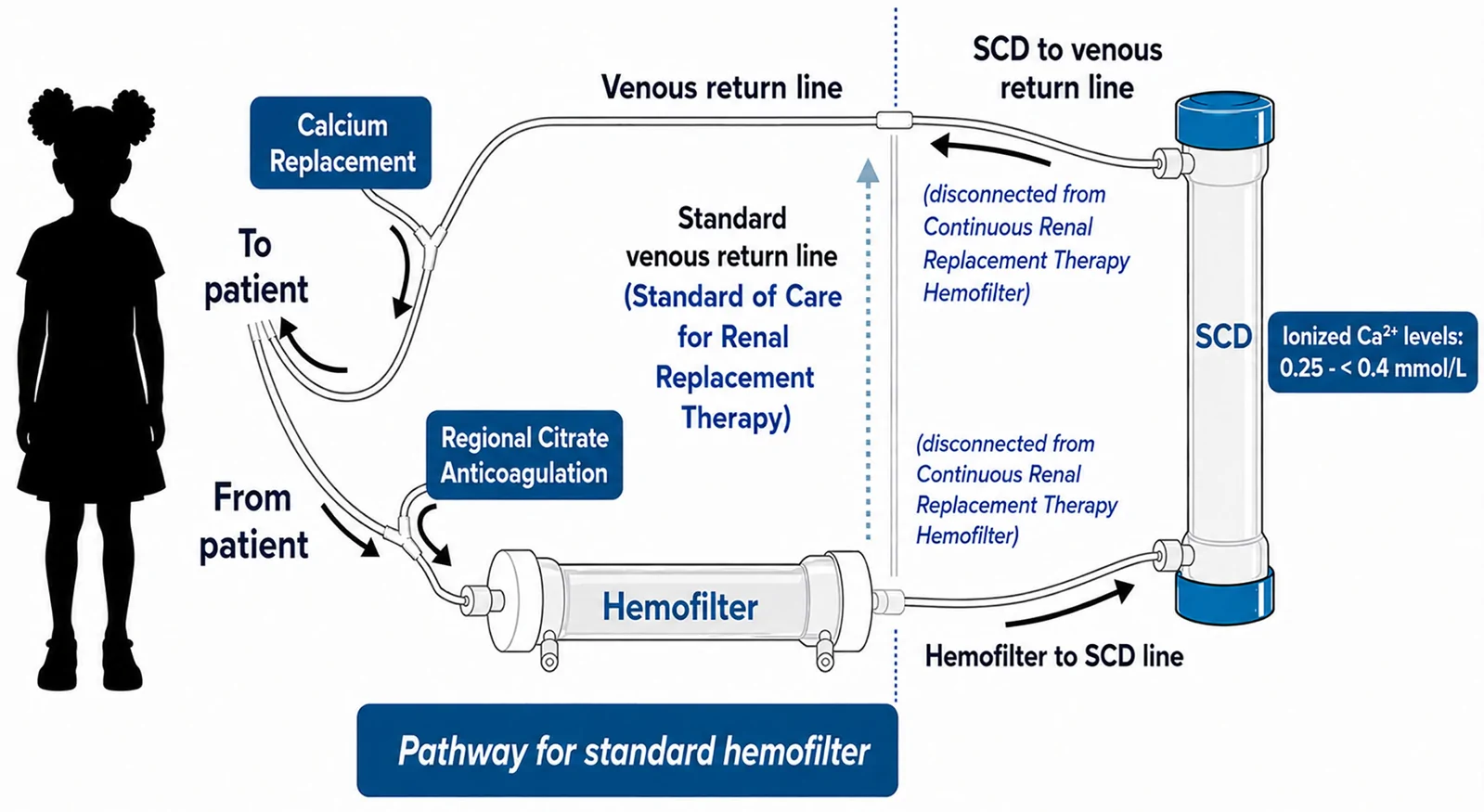
Continuous renal replacement therapy (CRRT) selective cytopheretic device (SCD) circuit configuration showing the blood flow path from the patient to the CRRT filter with the SCD-integrated post-CRRT filter. Ca^2+^ = calcium ion.

Despite enteral antimalarial therapy, she progressed to refractory shock with multiple organ failure, with a peak lactate of 21.9 mmol/L and parasitemia rising to 19% on HD 2. Given high parasite burden and severe *P. falciparum* infection, she underwent emergent RBC exchange transfusion and received her first dose of IV artesunate, reducing parasitemia to 9.9%. CRRT through right internal jugular venous access for refractory metabolic acidosis and oliguric AKI. The CRRT modality was continuous venovenous hemodiafiltration using a Prismaflex HF1000 filter (Vantive US Healthcare LLC, Deerfield, IL) and a prescription of 2000 mL/1.73 m^2^/hr with CRRT blood flow (mL/min): 170 mL/min. Given her profound hyperinflammatory state (**Supplemental Table 1**, https://links.lww.com/CCX/B679), she was simultaneously initiated on the SCD (SeaStar Medical, Denver, CO; **Fig. 1**) to modulate dysregulated innate immune activation. She remained on the SCD for 10 days and CRRT for 17 days before transition to intermittent hemodialysis.

Although parasite load declined rapidly, refractory shock and hyperlactatemia persisted. On HD 4, she developed progressive distal ischemia involving the right hand and subsequently bilateral toes and heels. The right radial arterial catheter was removed. Vascular imaging demonstrated preserved arterial flow without large-vessel occlusion. Hematologic evaluation revealed continued thrombocytopenia, reduced a disintegrin and metalloproteinase with a thrombospondin type 1 motif, member 13 activity (22.6%), and markedly elevated soluble C5b-9 (693 ng/mL), raising concern for secondary thrombotic microangiopathy. She received continuous fresh frozen plasma infusion followed by therapeutic plasmapheresis on HD 6, after which platelet counts stabilized and no new ischemic lesions developed, although existing areas progressed to dry gangrene.

She concurrently developed hepatic dysfunction with transaminitis and progressive hyperbilirubinemia with preserved synthetic function. Abdominal ultrasonography showed gallbladder wall edema without obstruction and splenomegaly. Inflammatory profiling revealed marked hyperinflammatory phenotype: C-reactive protein (26.4 mg/dL), ferritin (20,877 ng/mL), IL-6 (239 pg/mL), IL-18 (10,677 pg/mL), soluble IL-2 receptor (5,296 U/mL), and Chemokine [C-X-C motif] ligand 9 (30,786 pg/mL). Although systemic steroids, cytokine and complement inhibition were considered, these were deferred given improving clinical trajectory and concern for risks of immunosuppression. Hyperbilirubinemia peaked on HD 11 (total bilirubin 29.1 mg/dL; direct bilirubin 18.6 mg/dL), before gradually resolving.

Definitive antimalarial treatment included four doses of artemether-lumefantrine, eight doses of IV Artesunate, and three doses of atovaquone-proguanil. She was extubated on HD 15 with improved neurologic status and transferred to the general pediatric ward on HD 20 for continued renal support and rehabilitation. Peripheral blood smear (HD 29) confirmed complete parasitic clearance. Intermittent hemodialysis was discontinued (HD 33) following renal recovery. After prolonged hospitalization, she completed inpatient rehabilitation and was discharged home (HD 48) with improved hepatic function and resolution of systemic inflammation. At follow-up, organ dysfunctions had resolved, and she returned to baseline health with serum creatinine of 0.42 mg/dL and estimated glomerular filtration rate of 106.1 mL/min/1.73 m^2^.

Written informed consent was obtained for publication from the patient’s legal guardian.

## DISCUSSION

This patient presented with severe *P. falciparum* malaria complicated by cardiovascular, respiratory, renal, cerebral, hepatic, and hematologic failure, an often fatal clinical trajectory. Although parasite burden initiates disease, multiple organ dysfunction in SM is also driven by dysregulated host hyperinflammation, necessitating treatment of both parasitemia and the inflammatory cascade that propagates organ injury.

Initial therapy appropriately focused on rapid parasite destruction using RBC exchange and IV artesunate. Despite effective parasite reduction, the patient developed refractory metabolic acidosis and shock requiring escalating vasoactive and hemodynamic support. This progression mirrors the sepsis-like hyperinflammation phenotype observed in SM, in which neutrophil-endothelial activation promotes capillary leak, microvascular hypoperfusion, and tissue injury (28).

Given this pathophysiology, SCD therapy was initiated to directly modulate innate immune dysregulation. Under the low-ionized calcium conditions generated by regional citrate anticoagulation and the low-shear forces approximating capillary flow, SCD membranes selectively bind highly activated neutrophils and monocytes through calcium-dependent integrin interactions (29, 30). Bound neutrophils subsequently undergo deactivation and apoptosis and are naturally cleared by phagocytes of the reticuloendothelial system, restoring immune homeostasis rather than inducing immunosuppression (31, 32). Proinflammatory monocytes similarly shift toward a less inflammatory phenotype (32–34).

By attenuating neutrophil-endothelial activation, SCD therapy may reduce capillary sludging, tissue hypoxia, and toxic enzymatic injury, thereby disrupting the self-amplifying inflammatory cascade that drives organ dysfunction (28). Reduced proinflammatory monocyte activity may further promote transition of tissue macrophages toward reparative phenotypes (35), consistent with observations of improved recovery following severe AKI in SCD-treated patients (36).

In this patient, initiation of SCD therapy in combination with CRRT was temporally associated with declining inflammatory markers, particularly daily ferritin, and when measured IL-6, alongside reduced vasopressor requirements, and resolution of severe lactic acidosis. By HD 8, sustained net negative fluid balance during CRRT became achievable, suggesting reduced capillary leak, and coincided with improved pulmonary mechanics and oxygenation (Supplemental Table 1, https://links.lww.com/CCX/B679). Kidney injury peaked early and subsequently improved, consistent with mitigation of ongoing ischemic and inflammatory injury. These findings support the role of the SCD as an autologous leukocyte-processing system capable of modulating systemic hyperinflammation without inducing immunosuppression (21). Notably, discontinuation of SCD therapy on HD 11 while CRRT was continued was followed by an immediate rise in ferritin concentrations and absolute neutrophil count on HD 12, reversing prior improvements observed during combined SCD and CRRT therapy and further supporting a temporal association between SCD treatment and reduction in the patient’s inflammatory state beyond CRRT alone (**Supplemental Digital Content 1**, https://links.lww.com/CCX/B679).

Although the course was complicated by secondary thrombotic microangiopathy, hemodynamic stabilization and lactate improvement preceded complete parasite clearance, supporting the hypothesis that modulation of host response, rather than parasite clearance alone, was central to recovery. These observations reinforce that mortality from *P. falciparum* malaria infection is driven not solely by parasite burden but by a maladaptive host inflammatory response that amplifies endothelial dysfunction, metabolic failure, and multiple organ death (37, 38). Despite decades of investigation, no adjunctive immunomodulatory therapy has demonstrated consistent survival benefit in SM (39, 40). This first-in-human experience suggests that immune cell-directed extracorporeal therapy may represent a biologically targeted, nonimmunosuppressive adjunct therapy capable of addressing this long-standing therapeutic gap. The relatively low manufacturing cost of the SCD and easy integration into existing hemodialysis circuits make it a therapy that could be implemented in lower-income settings where malaria is prominent. Prospective clinical trials are warranted to evaluate SCD therapy as an adjunctive treatment for SM.

## Supplementary Material
