# Supplementary material for: Immunomodulation Therapy to Treat Severe Malaria With Multiple Organ Failure
Source: Crit Care Explor. 2026 Aug 27;8(9):e1471. doi: 10.1097/CCE.0000000000001471 (PMC13521499; doi:10.1097/CCE.0000000000001471)

Critical Care Explorations Supplemental Digital Content - Immunomodulation Therapy to Treat Severe Malaria with Multiorgan Failure

Table of Contents:

1. Supplemental Table 1: Physiologic parameters, vasoactive agents, therapy and treatment, and laboratory tests by hospital day for the first 13 days of the PICU admission (page 2-3)
2. Supplemental Figure 1: Trajectory of WBC, ANC, and Ferritin by Hospital Day (page 4)

Table 1. Physiologic parameters, vasoactive agents, therapy and treatment, and laboratory tests by hospital day for the first 13 days of the PICU admission.

**Physiologic Parameters (units)**	**HD 1**	**HD 2**	**HD 3**	**HD 4**	**HD 5**	**HD 6**	**HD 7**	**HD 8**	**HD 9**	**HD 10**	**HD 11**	**HD 12**	**HD 13**
MAP invasive (average, mmHg)	..	75	69	69	69	73	64	..	..	..	..	..	..
MAP non-invasive (average, mmHg)	68	69	81	64	69	80	68	73	66	59	69	72	88
PIP measured (max, cmH2O)	..	26	24	26	25	35	30	36	31	26	32	29	29
OI (max)	..	9	6	7	5	8	5	..	..	..	..	..	..
OSI (max)	..	..	..	..	..	..	6.3	10.4	9.8	7.1	8.2	8.7	2.6
Urine Output (mL)	..	684	1726	91	78	48	40	0	0	4	160	0	0
Weight (kg)	35.4	..	38.4	38.5	..	38.7	38.6	37.8	37.9	37.9	37	36.5	34.9
Net Fluid Balance (mL)	..	1454	244	34.9	411	151	750	-286	-745	-467	-58	-588	-364

**Vasoactive Agents (units)**													
Epi (max, mcg/kg/minute)	..	0.16	0.14	0.06	0.06	0.04	0.02	..	..	..	..	..	..
Vaso (max, milli-units/kg/minute)	..	3	1	2	..	..	..	..	..	..	..	..	..

**Therapy & Treatment**													
RBC Exchange Transfusion	..		..	..	..	..	..	..	..	..	..	..	..
CRRT with SCD	..											..	..
CRRT	..	..	..	..	..	..	..	..	..	..	..		
FFP Transfusions	..	..	..	..			..	..	..	..	..	..	..
Plasmapharesis	..	..	..	..	..		..	..	..	..	..	..	..
**Antimalarial Treatment:**													
Artemether–Lumefantrine	..		..	..	..	..		..	..	..	..	..	..
Artesunate	..						..	..			..	..	..

**Labs (units)**													
Parasitemia (max, %)	10.9	19	2.8	< 1	Negative	Negative	Negative	Negative	Negative	Negative	Negative	..	Negative
WBC (max, x10^3/mcL)	14.35	21.7	16.45	17.09	15.1	14.8	10.62	5.27	3.44	3.95	7.38	13.75	17.33
ANC (max, x10^3/mcL)	11.12	..	..	13.59	12.47	8.59	7.83	3.82	1.68	1.86	3.75	11.12	12.03
Hemoglobin (min, gm/dL)	6.8	7.8	9.2	8.4	8.4	8.5	9.6	10.6	10.3	10.5	10.7	10	9
Platelets (min, x10^3/mcL)	35	19	33	35	37	43	63	76	89	127	174	218	269
LDH (max, unit/L)	925	1838	2395	3065	3179	2750	1829	1769	1257	1176	1289	1390	1438
Haptoglobin (mg/dL)	<10	<10	<10	<10	<10	<10	<10	<10	<10	..	..	<10	..

BUN (max, mg/dL)	70	61	24	16	9	8	10	10	17	18	16	16	17
SCr (max, mg/dL)	1.6	1.33	0.53	0.55	0.71	0.82	0.85	0.99	0.93	0.96	0.88	0.92	0.85
NGAL (ng/mL)	..	649	5796	>18000	2058	1354	..	..	..	..	..	..	..
Albumin (min, gm/dL)	2.3	1.6	2.4	1.9	2.7	2.8	2.7	3.1	2.9	2.6	2.6	2.4	2.8
AST (max, unit/L)	185	285	854	959	937	789	500	502	292	196	152	133	124
ALT (max, unit/L)	87	62	167	224	245	231	154	197	162	143	131	115	93
Total Bilirubin (max, mg/dL)	4.5	3.5	3.9	5	7.9	9.4	12.6	22.9	25.2	26.8	29.7	29.4	28.1
PT (max, seconds)	10.9	14.7	15.1	12.5	11.7	12.2	13.2	12.3	..	..	..	..	..
INR (max)	1	1.35	1.39	1.15	1.07	1.12	1.21	1.13	..	..	..	..	..
APTT (max, seconds)	33.5	35.8	37.6	41	37.1	40.3	32.5	32.8	..	..	..	..	..
ADAMTS13 (% activity)	..	..	..	29.2	22.6	79.6	69.8	..	..	..	..	..	..
CXCL9 (pg/mL)	..	..	..	..	..	30786	..	..	..	..	..	..	16430
Procalcitonin (ng/mL)	61.54	..	..	..	..	..	..	..	..	..	..	..	..
CRP (mg/dL)	>10	..	20.3	25.7	23.7	22.1	18.4	20.7	23.5	26.4	26	17.8	10.4
Ferritin (ng/mL)	..	..	20877	17159	11815	7620	3851	3576	2308	3109	3600	7376	7118
IL-6 (pg/mL)	..	..	..	..	640	..	..	..	..	..	..	..	142
TNF alpha (pg/ml)	..	..	..	..	<2	..	..	..	..	..	..	..	2
Lactic Acid (max, mmol/L)	..	21.93	12.63	10.78	7.3	5.43	4.73	3.58	4.33	4.02	3.11	2.9	2.3

PICU = pediatric intensive care unit, HD = hospital day, MAP = mean arterial pressure, PIP = peak inspiratory pressure, OI = oxygenation index, OSI = oxygen saturation index, max = maximum, epi = epinephrine, vaso = vasopressin, RBC = red blood cell, CRRT = continuous renal replacement therapy, SCD = selective cytopheretic device, FFP = fresh frozen plasma, WBC = white blood cell, ANC = absolute neutrophil count, min = minimum, LDH = lactate dehydrogenase, BUN = blood urea nitrogen, SCr = serum creatinine, NGAL = neutrophil gelatinase-associated lipocalin, AST = aspartate aminotransferase , ALT = alanine aminotransferase, PT = prothrombin time, INR = international normalized ratio, APTT = activated partial thromboplastin time, CXCL9 = chemokine ligand 9, CRP = c-reactive protein, IL-6 = interleukin 6

Supplemental Figure 1

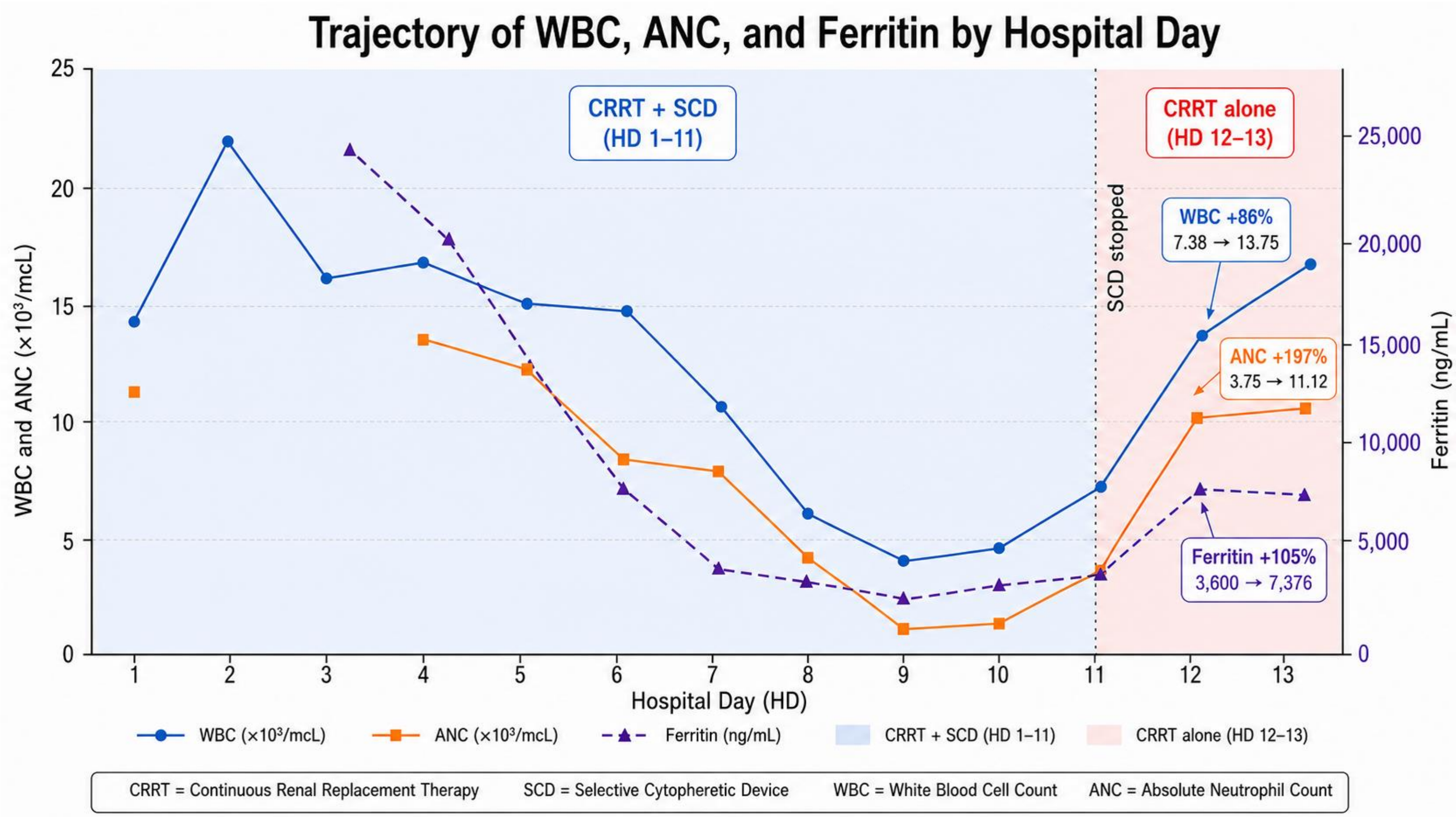

Supplement: Supplementary file 1 [file cc9-8-e1471-s001.pdf]
